# A novel amplification gene PCI domain containing 2 (PCID2) promotes colorectal cancer through directly degrading a tumor suppressor promyelocytic leukemia (PML)

**DOI:** 10.1038/s41388-021-01941-z

**Published:** 2021-10-08

**Authors:** Jingwan Zhang, Jianning Zhai, Chi Chun Wong, Huarong Chen, Xiaohong Wang, Jiafu Ji, Jun Yu

**Affiliations:** 1 Institute of Digestive Disease and Department of Medicine and Therapeutics, State Key Laboratory of Digestive Disease, Li Ka Shing Institute of Health Sciences, Shenzhen Research Institute, The Chinese University of Hong Kong, Hong Kong, China; 2 Department of Gastrointestinal Oncology, Peking University Cancer Hospital & Institute, Beijing, China

**Keywords:** Colorectal cancer, Predictive markers

## Abstract

Using whole genome sequencing, PCI Domain Containing 2 (*PCID2*) was identified to be amplified in colorectal cancer (CRC). In this study, we investigated the expression, biological function, molecular mechanism, and clinical implication of PCID2 in CRC. PCID2 mRNA and protein expression were higher in CRC cells and tumor tissues compared to healthy colonic tissues. The copy number of PCID2 was positively correlated with its mRNA expression. Multivariate analysis revealed that PCID2 is an independent prognostic factor for CRC recurrence. Functional studies showed that PCID2 promoted cell growth, cell cycle progression, and cell migration/invasion, while apoptosis was suppressed. Moreover, PCID2 promoted xenograft growth and lung metastasis in nude mice. Using co-immunoprecipitation and mass spectroscopy, we showed that PCID2 binds to promyelocytic leukemia (PML), a tumor suppressor involved in non-canonical β-catenin signaling. PCID2 promoted the degradation of PML via poly-ubiquitination, which in turn, induced Wnt/β-catenin signaling while simultaneously repressing ARF-p53 pathway. Thus, these results demonstrated that PCID2 functions as an oncogene in CRC by enhancing canonical Wnt/β-catenin signaling and inhibition of CTNNB1-ARF-p53 axis. PCID2 promoted canonical Wnt/β-catenin signaling in CRC via degradation of PML. PCID2 may serve as an independent prediction marker for CRC recurrence.

## Introduction

Colorectal cancer is the second most common cancer in females and the third most common cancer in males [1]. Each year, over 1.2 million cases are diagnosed with about 600,000 deaths, making CRC the fourth most common cause of cancer-related deaths worldwide [2]. Surgical resection is the best treatment option for patients with CRC, but tumor recurrence after resection, both local and distant, is associated with poor prognosis [3]. Colorectal carcinogenesis is a multi-step process that involves consecutive genetic and epigenetic alterations [4]. Copy number alterations (CNAs) are a form of structural genetic variation associated with a gain and loss of whole or part of chromosomes. CNAs contribute to tumorigenesis through activation of driver oncogenes and inactivation of tumor suppressor genes [5–7]. CNAs in p53, APC or KRAS are key events in tumorigenesis and metastatic progression [8–10]. Hence, the identification of novel genes with CNAs may reveal novel oncogenic mechanisms in colorectal carcinogenesis.

Chromosome 13q34 is one of the most frequently amplified regions in CRC and it contains several putative oncogenes [11–13]. Using whole genome sequencing, we identified PCI domain containing 2 (*PCID2*) as a novel gene amplified at 13q34 in cancer. PCID2, also known as CSN12-Like Protein, is regarded as a homolog of the yeast Thp1 based on their structural similarity. However, the role of PCID2 in CRC is largely unexplored. We, therefore, hypothesized that PCID2 amplification could be an important mechanism for colorectal carcinogenesis. In this study, we aim to investigate the amplification status of PCID2, characterize its function and molecular mechanism, and evaluate the clinical impact of PCID2 in CRC. We identified that PCID2 amplification drives its overexpression in CRC, and PCID2 high expression was associated with CRC recurrence. Functional studies showed that PCID2 functions as an oncogenic factor in CRC. Mechanistically, PCID2 physically associated with Promyelocytic leukemia protein (PML), the gene has been demonstrated to be involved in the development of acute promyelocytic leukemia (APL) [14]. PML was also reported to be a tumor suppressor in many cancer types [15]. Furthermore, it has been approved that PML, p300, and beta-catenin cooperated in transactivation of a subset of beta-catenin-responsive genes including ARF and Siamois but not Cyclin D1 [16]. PCID2 induced degradation of PML, which reduced noncanonical Wnt/β-catenin signaling (CTNNB1-ARF-p53), whilst promoting canonical Wnt/β-catenin signaling to promote oncogene expression (c-Myc, Cyclin D1, etc.). Our results revealed the molecular mechanism and the clinical application of PCID2, a novel oncogene, in CRC.

## Results

### PCID2 is overexpressed in primary colorectal tumors

**Fig. 1:**
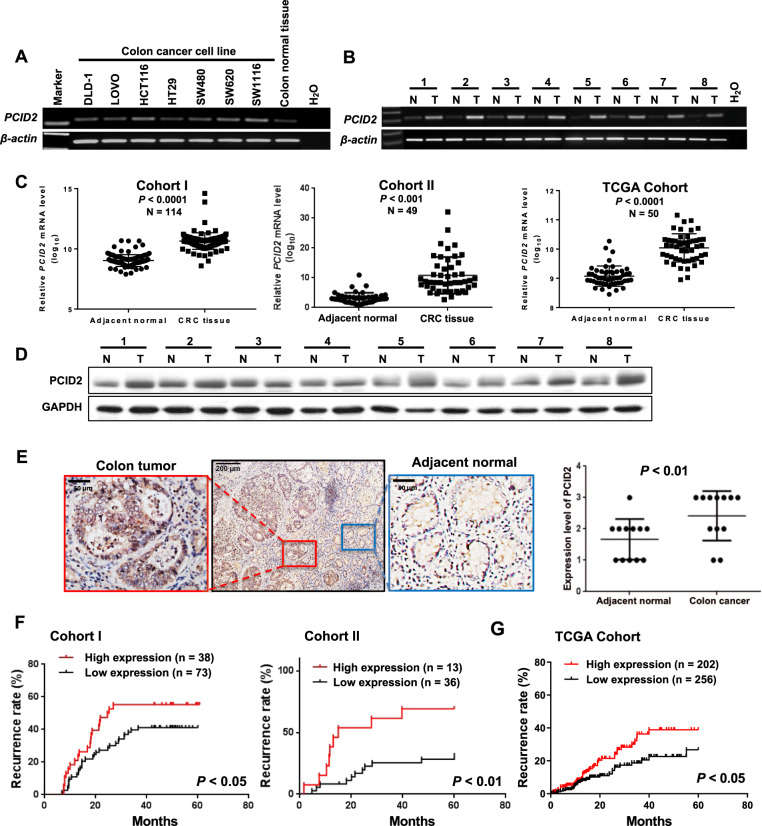
PCID2 is overexpressed in CRC cell lines and primary tissues. **A** PCID2 mRNA was expressed in CRC cell lines, but not in normal colon tissues, as determined by RT-PCR. **B** mRNA expression of PCID2 was significantly higher in primary CRC tumors compared to their adjacent tissues. **C** Higher PCID2 mRNA expression was observed in CRC compared to paired adjacent normal tissues as shown by qRT-PCR (Left: Cohort I; Middle: Cohort II; Right: TCGA cohort). **D** Protein expression of PCID2 was significantly higher in primary CRC tumors compared to their adjacent tissues by western blot. **E** PCID2 protein expression was significantly higher in primary CRC compared to adjacent normal tissues as shown by IHC staining. **F** Disease-free survival in relation to PCID2 expression was evaluated by the Kaplan–Meier survival curve. CRC patients with high PCID2 expression had higher recurrence rate than those with low PCID2 expression. **G** The predictive value of PCID2 expression in CRC recurrence was validated in CRC cohort from TCGA study. Two-tailed paired *t* test, log-rank test.

PCID2 expression was examined in CRC cells and colorectal tumor tissues. Compared to normal colon tissues, PCID2 mRNA was highly expressed in CRC cell lines as determined by PCR analysis (Fig. 1A). Moreover, PCID2 mRNA was significantly increased in CRC tissues compared with paired adjacent normal tissues in both Cohort I and Cohort II (*P* < 0.001 and *P* < 0.01) and TCGA dataset (*P* < 0.0001) (Fig. 1B, C). In keeping with the increased PCID2 mRNA, PCID2 protein expression was significantly higher in CRC tumors as compared to paired adjacent normal tissues, as evidenced by Western blot (*n* = 8) and IHC (*n* = 12) analysis (Fig. 1D, E). These results indicated the aberrant up-regulation of PCID2 in CRC.

### PCID2 is an independent predictor of cancer recurrence in patients with CRC

We next evaluated the association of PCID2 expression with the clinicopathological features and clinical outcomes of CRC patients. As shown in Table 1, there was no correlation between PCID2 expression and age, gender, tumor localization of patients with CRC. In univariate Cox regression analysis of Cohort I and Cohort II, high PCID2 expression was associated with an increased risk of metastasis-related recurrence (Cohort I: RR 1.145, 95% CI, 1.036 to 1.265; *P* = 0.008; *P* = 0.008; Cohort II: RR 2.077, 95% CI, 1.082–3.988), especially for stage II and stage III patients in Cohort II (Table 1). After adjusting for potential confounding factors, multivariate Cox regression analysis showed that PCID2 overexpression was an independent predictor of recurrence in CRC patients (Cohort I: RR 1.140, 95% CI, 1.035–1.255; *P* = 0.008; Cohort II: RR 2.095, 95% CI, 1.089–4.031; *P* = 0.013) (Table 1). Recurrence curves showed that the patients with CRC with high PCID2 expression had increased risk of recurrence compared with those with low PCID2 (*P* < 0.05 for Cohort I and *P* < 0.01 for Cohort II, log-rank test) (Fig. 1F). Our findings were validated in TCGA cohort, where PCID2 was confirmed to be independently associated with CRC recurrence (Supplementary Table 4). CRC patients with overexpression of PCID2 had higher risk of cancer recurrence (Fig. 1G, *P* < 0.05). Since advanced stage of CRC is more related with cancer recurrence, we further investigated the expression of PCID2 in different stages of CRC. As shown in Supplementary Fig. 1, no significant differences of PCID2 expression at different stages of CRC observed in Cohort I and TCGA Cohort. Taken together, these findings indicated that high expression of PCID2 is an independent factor predicting CRC recurrence.

**Table 1:** Cox regression analysis of potential recurrence predictor for patients with colon cancer.

Variables	Univariate			Multivariate	
	RR (95% CI)	*P* value		RR (95% CI)	*P* value
**Corhort I**					
Age	1.007 (0.986–1.028)		0.527	1.003 (0.982–1.025)	0.771
Gender					
M	1.098 (0.618–1.950)		0.749	0.808 (0.450–1.453)	0.477
F	1			1	
T stage					
II	1.978 (0.712–5.493)		0.191		
III	1				
Localization					
Colon	1.065 (0.614–1.849)		0.823		
Rectum	1				
Differentiation					
Moderate/High	1.159 (0.606–2.215)		0.656		
Low	1				
PCID2 expression					
High	1.145 (1.036–1.265)		0.008	1.140 (1.035–1.255)	0.008
Low	1			1	
**Corhort II**					
Age	0.655 (0.278–1.544)		0.333	1.983 (0.747–5.266)	0.17
Gender					
M	1.630 (0.632–4.203)		0.312	2.748 (0.950–7.950)	0.162
F	1			1	
TNM					
I	0		0.972	0	0.973
II	0.266 (0.092–0.733)		0.01	0.198 (0.063–0.7617)	0.005
III	1		0.038	1	0.02
Localization					
Colon	1.780 (0.238–13.307)		0.574		
Rectum	1				
PCID2 expression					
High	2.077 (1.082–3.988)		0.008	2.095 (1.089–4.031)	0.013
Low	1			1	

### Amplification of PCID2 contributes to its overexpression in CRC tumor tissues

**Fig. 2:**
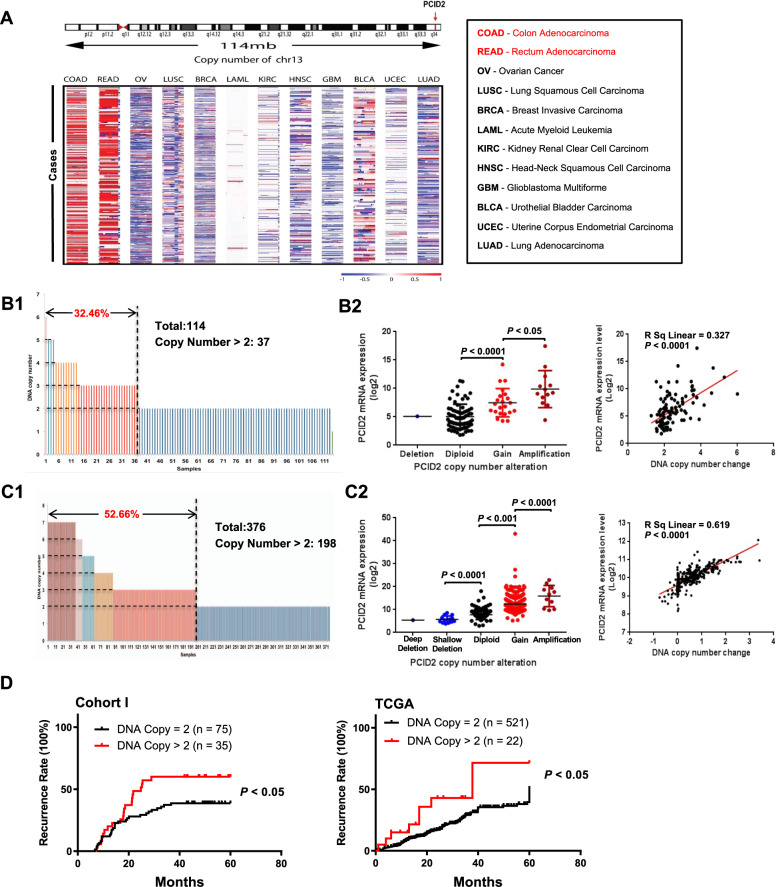
Overexpression of PCID2 is due to copy number gain in colorectal cancer. **A** Copy number variations of 13q14 across 12 human cancer types in the TCGA pan-cancer dataset. PCID2 showed copy number gain specifically in colon and rectal cancers. Red, copy number gain; blue, copy number loss. **B** The association of PCID2 gene amplification and mRNA expression was determined in 114 CRC tissues from cohort I. **B1** PCID2 was frequently amplified in CRC. PCID2 DNA copy number was examined using a PCID2-specific probe by genomic DNA real-time PCR. **B2** PCID2 copy number was positively correlated with its mRNA expression (*n* = 114; *P* < 0.0001, Pearson correlation coefficient analysis). **C** The association of PCID2 gene amplification and mRNA expression was validated in 376 CRC tissues from TCGA database. **C1** PCID2 was frequently amplified. **C2** PCID2 copy number was positively correlated with its mRNA expression (*n* = 376; *P* < 0.0001, Pearson correlation coefficient analysis). **D** Kaplan–Meier recurrence risk analysis based on PCID2 copy number status in Cohort I and TCGA database. PCID2 copy number amplification was associated with a greater risk of recurrence in patients with colorectal cancer. Two-tailed unpaired *t* test, log-rank test.

PCID2 is localized at chromosome 13q34, which is a highly amplified region in CRC. We evaluated the copy number variations (CNVs) in TCGA pan-cancer dataset. PCID2 was amplified in CRC among all 12 cancer types (Fig. 2A and Supplementary Fig. 2). Thus, we speculated that high expression of PCID2 in CRC was a consequence of gene amplification. To validate our hypothesis, DNA copy number of PCID2 was determined by genomic DNA quantitative PCR (Life technologies) in Cohort I. PCID2 was amplified in 32.5% (37/114) of all cases (Fig. 2B1). Moreover, PCID2 mRNA expression was significantly higher in CRC with copy number amplification, with PCID2 mRNA showing a positive correlation with its DNA copy number (Fig. 2B2, *R*^2^ = 0.327; *P* < 0.0001). These results were further validated in TCGA cohort. Gene amplification of PCID2 was found in 52.7% (198/376) of primary CRC (Fig. 2C1), and increased PCID2 copy number positively correlated with its mRNA expression (Fig. 2C2, *R*^2^ = 0.619; *P* < 0.0001). Furthermore, PCID2 gene amplification (DNA copy number >2) was associated with increased risk of recurrence in CRC cohort I (*P* < 0.05) and TCGA cohort (*P* < 0.05) (Fig. 2D). These data suggested that gene amplification could be the major mechanism leading to PCID2 overexpression in CRC.

### PCID2 promotes colorectal cancer cell proliferation in vitro

**Fig. 3:**
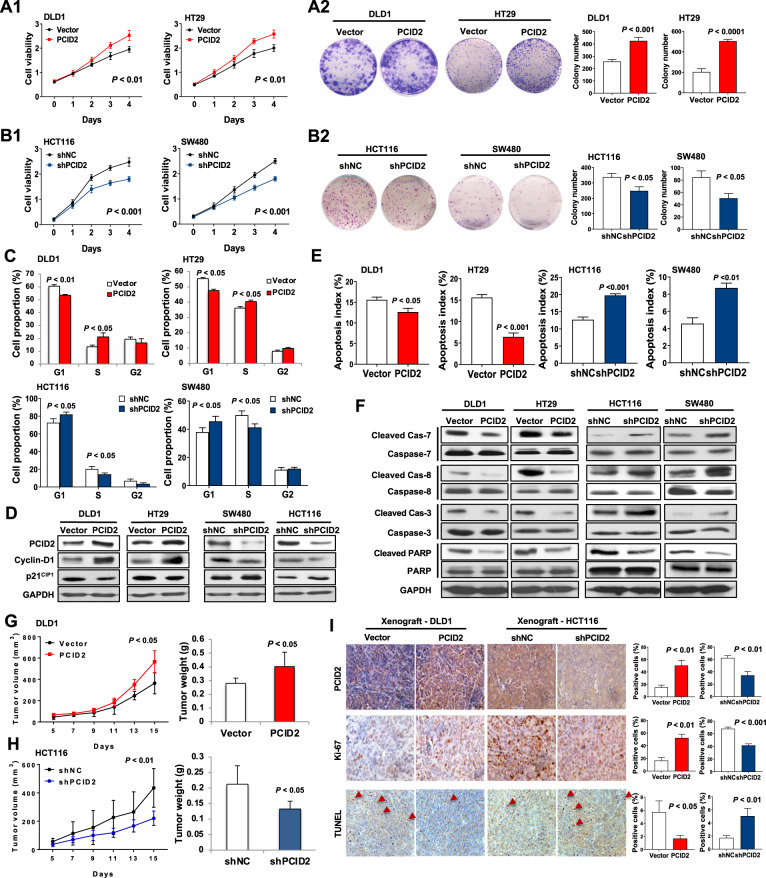
PCID2 promotes colorectal cancer cell growth and induces tumorigenesis in vivo. **A** PCID2 increased cell proliferation in DLD1 and HT29 cells. Cell growth (**A1**) and colony formation (**A2**) was promoted by ectopic expression of PCID2. **B** PCID2 knockdown inhibited cell growth (**B1**) and colony formation (**B2**) in HCT116 and SW480 cells. **C** PCID2 overexpression promoted cell cycle transition from G1 to S phase, while PCID2 knockdown showed the opposite effect. **D** PCID2 overexpression increased protein levels of Cyclin D1 and p21^CIP1^, while an opposite effect was observed after PCID2 knockdown. **E** Ectopic expression of PCID2 suppressed cell apoptosis, while PCID2 knockdown promoted cell apoptosis as determined by Annexin V/7-AAD staining and flow cytometry. **F** PCID2 reduced protein expression of the cleaved forms of caspase-8, caspase-7, caspase-3, and caspase-PARP by Western blot analysis, while PCID2 knockdown promoted their expression. **G** PCID2 overexpression increased tumor volume and weight of DLD1 subcutaneous xenografts in nude mice. **H** PCID2 knockdown repressed tumor volume and weight of HCT116 subcutaneous xenografts in nude mice. **I** IHC staining confirmed PCID2 overexpression in DLD1 xenografts and PCID2 knockdown in HCT116 xenografts. Effect of PCID2 on cell proliferation and apoptosis was determined by Ki-67 and TUNEL staining, respectively. Two-tailed paired *t* test, ANOVA test.

A series of in vitro assays were performed with gain- or loss-of-function of PCID2. DLD1 and HT29 cells with low PCID2 expression were transfected with PCID2 or control vector, whereas PCID2-targeting shRNA and corresponding controls were used to knockdown PCID2 in HCT116 and SW480 cells (Supplementary Fig. 3). Ectopic PCID2 expression promoted cell viability and clonogenicity as compared to controls, as determined by cell viability and colony formation assays, respectively (Fig. 3A1, A2). Conversely, PCID2 knockdown in HCT116 and SW480 cells inhibited CRC cell growth and clonogenicity (Fig. 3B1, B2).

### PCID2 promotes cell cycle progression and suppresses cell apoptosis

To determine the mechanism by which PCID2 promoted cell growth, we analyzed the effect of PCID2 on cell cycle distribution by flow cytometry. PCID2 induced G1-S cell cycle phase transition in CRC cell lines (Fig. 3C). Consistently, PCID2 induced protein expression of G1-S transition promoter cyclin D1 but inhibited the expression of G1 gatekeeper p21^Cip1^ (Fig. 3D). These results suggested that PCID2 acts on the G1/S checkpoint to promote cell cycle progression in CRC cells. We next determined the effect of PCID2 on cell apoptosis. PCID2-overexpressing DLD1 and HT29 cell lines demonstrated decreased numbers of apoptotic cells compared to controls (Fig. 3E). In contrast, the proportion of apoptotic cells was significantly increased in PCID2 shRNA-transfected HCT116 and SW480 cells compared to control shRNA-transfected cells (Fig. 3E). Protein levels of key cell apoptosis regulators including cleaved forms of capase-7, caspase-8, caspase-3 and PARP were decreased significantly by PCID2 overexpression in DLD1 and HT29 cells, while they were induced in HCT116 and SW480 cells with PCID2 knockdown (Fig. 3F). These results indicate that PCID2 suppresses apoptosis in CRC cells. Consistent with these data, Cancer Pathway PCR Array analysis of shNC- and shPCID2-expressing HCT116 cells revealed that PCID2 knockdown significantly altered the expression of genes involved in cell cycle and apoptosis (Supplementary Fig. 4).

### PCID2 promotes tumorigenicity in vivo

Based on our in vitro results, we examined the effect of PCID2 on tumorigenicity in vivo. We stably overexpressed and silenced PCID2 in DLD1 and HCT116 cells, respectively. DLD1-PCID2 tumor xenografts grew significantly faster than DLD1-vector control xenografts, with higher tumor weight at end point (Fig. 3G and Supplementary Fig. 5A). On the contrary, HCT116-shPCID2 tumors showed significantly longer latency and reduced tumor volume compared to controls (Fig. 3H and Supplementary Fig. 5A). PCID2 expression in xenograft tumors were confirmed by RT-PCR, Western blot (Supplementary Fig. 5B) and IHC staining (Fig. 3I). PCID2 promoted tumor growth in vivo through inducing cell proliferation and inhibiting apoptosis, as evidenced by Ki-67 and TUNEL staining, respectively (Fig. 3I). Together, our data validated the pro-tumorigenic role of PCID2 in vivo.

### PCID2 promotes tumor invasion in vitro and metastasis in vivo

**Fig. 4:**
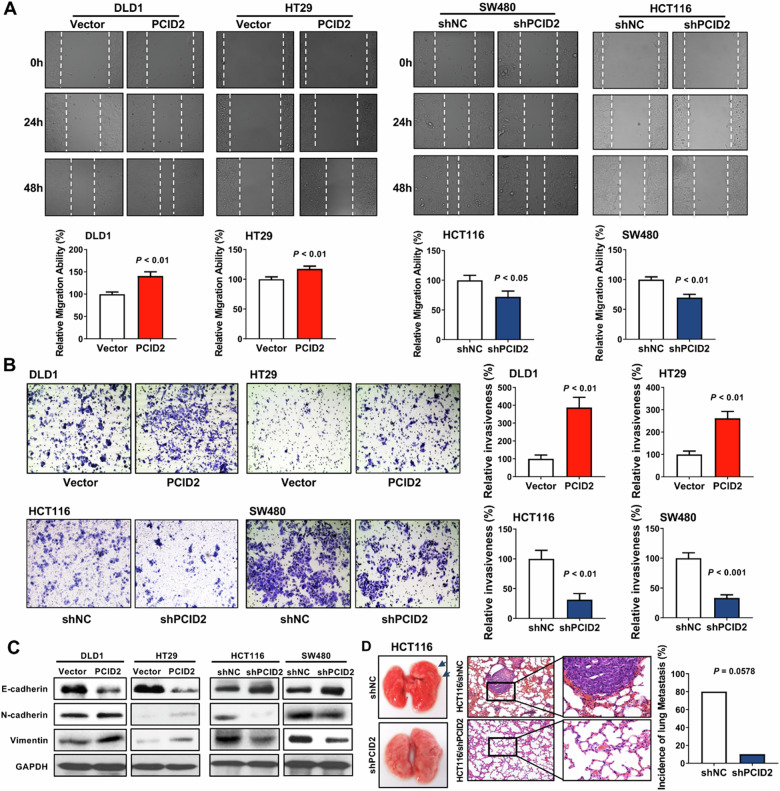
PCID2 promotes pro-metastatic properties in vitro and in vivo. **A** Overexpression and knockdown of PCID2 significantly increased and decreased migration ability in CRC cells, respectively. **B** Ectopic expression of PCID2 promoted cell invasion in DLD1 and HT29 cells. Knockdown of PCID2 suppressed cell invasion in HCT116 and SW480 cells. **C** PCID2 increased the expression of mesenchymal markers (N-cadherin and vimentin) and decreased the expression of epithelial marker E-cadherin. PCID2 knockdown exerted the opposite effects. **D** PCID2 promoted lung metastasis of HCT116 cells in nude mice after tail vein injection. Two-tailed unpaired *t* test.

Given that PCID2 was associated with CRC recurrence, we investigated the effect of PCID2 on pro-metastatic ability of CRC cells. Wound healing and Matrigel invasion assays revealed that PCID2 overexpression increased both migratory and invasive abilities of DLD1 and HT29 cells (Fig. 4A, B). In contrast, PCID2 knockdown inhibited cell migration and invasion in HCT116 and SW480 cells (Fig. 4A, B). In keeping with this, PCID2 overexpression promoted epithelial-to-mesenchymal transition (EMT) markers, as evidenced by increased expression of N-cadherin and Vimentin but decreased expression of E-cadherin (Fig. 4C). On the contrary, PCID2 knockdown reversed the EMT phenotype (Fig. 4C). Cancer Pathway PCR array further validated the role of PCID2 in modulating expression of genes associated with cell adhesion, angiogenesis, migration, and invasion (Supplementary Fig. 4).

We next performed experimental lung metastasis model by injecting nude mice with HCT116-shNC or HCT116-shPCID2 cells via the tail vein. As shown in Fig. 4D, increased incidence and multiplicity of metastasis nodules were observed in lung tissues collected from HCT116-shNC injected group (4/5, 80%) compared to HCT116-shPCID2 group (1/5, 20%). These results indicated that PCID2 promotes pro-metastatic properties of CRC cells in vitro and in vivo.

### PML is a direct interacting partner of PCID2

**Fig. 5:**
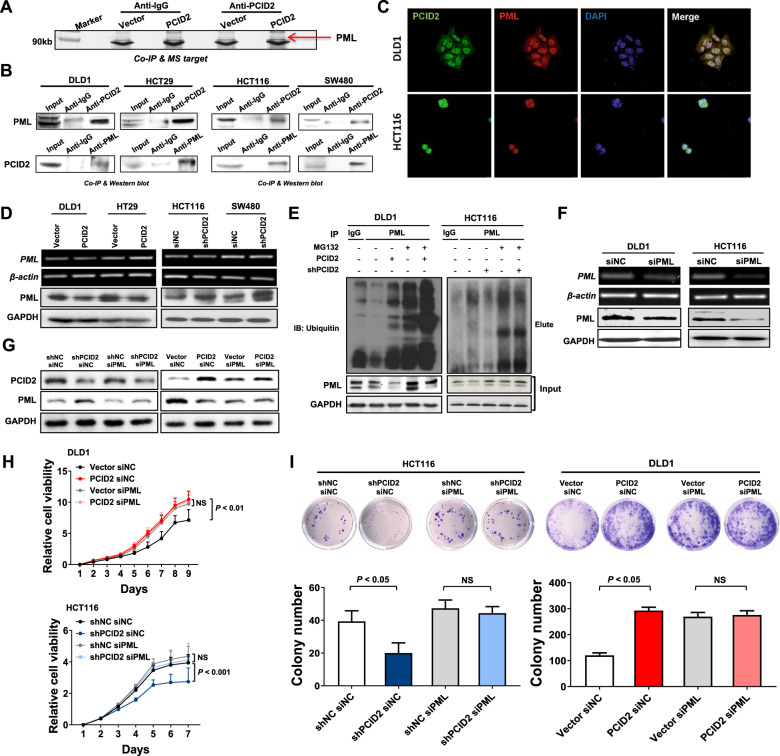
PCID2 interacts with promyelocytic leukemia (PML) by mediating the degradation of PML. **A** Immunoprecipitant of PCID2 was analyzed by sodium dodecyl sulfate-polyacrylamide gel electrophoresis (SDS-PAGE) and proteins were stained by silver staining. Proteins of interest were identified by mass spectrometry (indicated by arrow). **B** The interaction between PCID2 and PML was confirmed by co-immunoprecipitation (IP). **C** PCID2 and PML are co-localized in the nucleus as demonstrated by confocal immunofluorescence microscopy. **D** Ectopic expression or knockdown of PCID2 did not alter mRNA expression of PML, but decreased and enhanced protein expression of PML, respectively. **E** Ectopic expression of PCID2 promoted polyubiquitination of PML. Cell lysates were subjected to immunoprecipitation (IP) with anti-PML antibody or rabbit IgG as control, followed by immunoblotting with respective antibodies as indicated. Smear bands corresponds to polyubiquitinated PML. **F** Knockdown of PML in DLD1 and HCT116 cells was confirmed by RT-PCR and Western blot. **G** Protein expression of PCID2 and PML were checked by Western blot. **H** Knockdown of PML abolished the growth-promoting effect mediated by PCID2 determined by cell viability assay. **I** Knockdown of PML significantly abolished the promoting effect of PCID2 on clonogenicity. Two-tailed unpaired *t* test, NS not significant.

To elucidate the molecular mechanism of PCID2, we performed immunoprecipitation followed by mass spectrometry analysis to identify the potential interacting partners of PCID2 in CRC. PCID2 binding candidates were recognized by bands that were specifically found in PCID2 overexpressing cells (Fig. 5A). Among them, PML is one candidate of interest due to its nuclear localization and involvement in Wnt signaling pathway [16] (Fig. 5A). To confirm the interaction of PCID2 with PML, co-immunoprecipitation was performed. As expected, a positive PML signal was observed in the immunoprecipitant pulled down by anti-PCID2 antibody. Reciprocal co-immunoprecipitation by anti-PML antibody also resulted in the detection of PCID2 (Fig. 5B). Furthermore, confocal microscopy showed that PCID2 and PML were co-localized in the nucleus of DLD1 and HCT116 cells (Fig. 5C). These results implied direct physical interaction and nuclear co-localization of PCID2 and PML in CRC cells.

### PCID2 induces PML protein degradation via polyubiquitination

We next evaluated the interplay between PCID2 and PML. As shown in Fig. 5D, PML mRNA was not significantly altered by PCID2 overexpression (DLD1 and HT29) or knockdown (HCT116 and SW480). However, PML protein expression was negatively regulated by PCID2 overexpression, whereas PCID2 knockdown increased PML protein levels (Fig. 5D). Moreover, we found that PCID2 mRNA expression level was negatively correlated with PML protein expression in online cohort (TCGA cohort) (Supplementary Fig. 6). We therefore hypothesized that PCID2 might mediate PML protein degradation. Previous work showed that PML is primarily degraded via the Ubiquitin/Proteasome system (UPS) [17, 18]. To prove this conjecture, we treated cells with MG132, an inhibitor of UPS. Indeed, PML protein expression was restored in PCID2-expressing DLD1 cells treated with MG132 (Fig. 5E), implying that PCID2-induced degradation of PML depends on the UPS. Treatment of PCID2 silenced HCT116 cells with MG132, however, did not further increase PML protein expression (Fig. 5E). To confirm the involvement of UPS, we examined polyubiquitination of PML, a marker of UPS-mediated protein degradation. PCID2 dramatically promoted polyubiquitination of the endogenous PML, whereas knockdown of PCID2 inhibited polyubiquitination of PML (Fig. 5E). Collectively, these data suggested that PCID2 promotes PML degradation through promoting polyubiquitination.

### The oncogenic function of PCID2 is dependent on PML

As PCID2 interacts with PML and promotes PML degradation, we next asked if PML is involved in the oncogenic effect of PCID2. We first detected the PML siRNA transfection efficiency in HCT116 and DLD1 cell lines (Fig. 5F). Furthermore, PML siRNA was transfected into DLD1 and HCT116 cells with the ectopic expression and knockdown of PCID2, respectively. The expression of PCID2 and PML were further checked (Fig. 5G). In HCT116 cells with silenced PCID2, PML knockdown restored cell viability (Fig. 5H) and colony formation (Fig. 5I). In DLD1 cells, siPML increased cell viability in control cells, and more importantly, nullified the growth promoting effect of PCID2 overexpression (Figs. 5H, I). We further investigated the relationship between PML expression and CRC recurrence, we found that high expression of PML was associated with CRC recurrence (Supplementary Fig. 7). Hence, PCID2-mediated oncogenic function in CRC is dependent on PML.

### PCID2 activates Wnt/β-catenin signaling in CRC

**Fig. 6:**
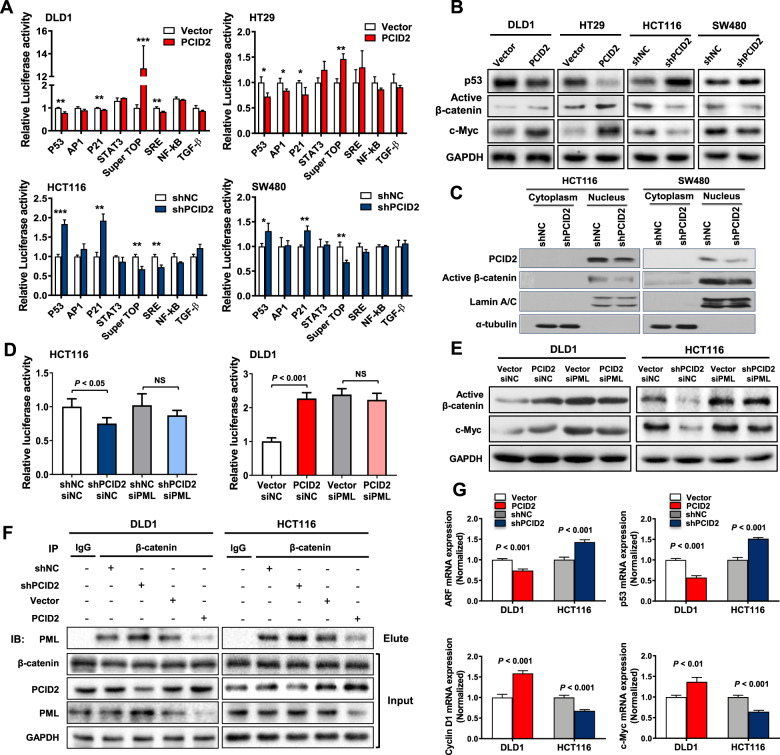
PCID2 mediated the degradation of PML, resulting in the bi-directional regulation of canonical and non-canonical Wnt signaling pathways. **A** Luciferase reporter assay for eight major cancer-related pathways was performed in PCID2-overexpressed and PCID2-silenced CRC cells. **B** Protein expression of active β-catenin, c-Myc and p53 was determined by Western blot. **C** PCID2 is localized in the nucleus and PCID2 knockdown reduced the expression of active β-catenin as demonstrated by Western blot of cytoplasmic and nuclear fractions. **D**, **E** Knockdown of PML abrogated the effect of PCID2 on activation of Wnt signaling pathway. **F** The interaction between PML and β-catenin was determined by IP assay. Depletion and overexpression of PCID2 increased and decreased the binding of PML to β-catenin, respectively. **G** mRNA expression of downstream effectors of non-canonical β-catenin signaling (*ARF* and *p53*) and canonical Wnt/β-catenin signaling (*Cyclin D1* and *c-Myc*) was determined by qPCR. Two-tailed paired *t* test, NS not significant, *P < 0.05, **P < 0.01, ***P < 0.001.

To gain insights into the molecular basis of the oncogenic effect of PCID2 in CRC, we first screened eight molecular pathways in CRC by luciferase report assay. We noticed that Wnt signaling pathway (TOP-flash) was enhanced, while p53 and p21 pathways were inhibited after ectopic expression of PCID2 in HT29 cells and DLD1 cells (Fig. 6A). On the contrary, knockdown of PCID2 exerted opposite effects on these pathways (Fig. 6A). Consistently, overexpression of PCID2 upregulated active-β-catenin and c-Myc and downregulated p53, while PCID2 knockdown had a reverse effect on these markers (Fig. 6B). Moreover, shPCID2 also reduced the levels of cytoplastic β-catenin and nuclear active β-catenin (Fig. 6C and Supplementary Fig. 8), suggesting that Wnt/β-catenin signaling is the key downstream pathway activated by PCID2. We next questioned if the effect of PCID2 on Wnt/β-catenin signaling is dependent on PML. We performed siPML-mediated knockdown in DLD1 and HCT116 cells with the ectopic expression and knockdown of PCID2, respectively, followed by TOP-flash assay for Wnt signaling activity. As shown in Fig. 6D, PCID2 overexpression failed to induce TOP-flash in DLD1 cells with silenced PML, whereas siPML restored TOP-flash activity in PCID2-depeted HCT116 cells. Consistent with this, silencing of PML promoted active β-catenin and c-Myc in control DLD1 cells, but attenuated the effect of PCID2 on these Wnt activation markers in DLD1-PCID2 cells (Fig. 6E). Similarly, PML knockdown restored active β-catenin and c-Myc expression in PCID2-silenced HCT116 cells (Fig. 6E). These results indicated that PCID2 may promote tumorigenesis through activation of Wnt signaling, and effect that is dependent on PML.

### PCID2 suppresses noncanonical Wnt signaling pathway through PML degradation

PML forms a complex with β-catenin and p300 to transactivate of a subset of β-catenin-responsive genes including *ARF*, but not oncogenic factors including Cyclin D1 and c-Myc [16]. We reasoned that PML might promote alternative activation of β-catenin via a β-catenin-ARF-p53 axis, thereby suppressing its involvement in canonical Wnt/β-catenin signaling. To investigate this hypothesis, we performed immunoprecipitation assay to identify the interaction between PML and β-catenin. We found that PML immunoprecipitated with β-catenin was decreased upon PCID2 overexpression in DLD1 cells, while a reverse trend was observed after PCID2 knockdown in HCT116 cells (Fig. 6F). Consistently, expression of downstream targets of non-canonical β-catenin-related signaling, ARF and p53, were decreased by PCID2 overexpression, but were induced by PCID2 knockdown in CRC cell lines (Fig. 6G). These results illustrated that PML can activate the tumor suppressive noncanonical β-catenin-related signaling via competitive binding with β-catenin.

## Discussion

In this study, we revealed PCID2 as a novel amplification gene that is overexpressed in CRC. PCID2 is located at 13q34, a genomic region with a higher frequency of amplification in CRC compared to other common cancer types [11–13]. Analysis of independent CRC cohorts demonstrated that PCID2 copy number gain occurs in 30–50% of CRC patients. PCID2 amplification is positively associated with its mRNA expression in CRC patients from our cohort and TCGA dataset, implying that copy number gain contributes to high PCID2 expression in colorectal carcinogenesis. Moreover, PCID2 high expression is an independent predictor for recurrence-free survival in CRC patients. Hence, we hypothesize that PCID2 might be a potential oncogenic factor in CRC.

A series of in vitro and in vivo assays demonstrated that PCID2 possesses pro-tumorigenic function. Ectopic expression of PCID2 in CRC cell lines induced cell proliferation in vitro and tumorigenesis in nude mice; while PCID2 knockdown exerted opposite effects. PCID2 promoted CRC cell growth is mediated by accelerating G1-S cell-cycle transition and inhibition of apoptosis. PCID2 induced G1-S transition is associated with the up-regulation of cyclin D1 and down-regulation of p21Cip1, two key regulators of the transition from G1 to S phase of the cell cycle [19–21]. Concomitantly, cell growth promoted by PCID2 was also related to inhibition of caspase-dependent apoptosis [22, 23]. Besides, PCID2 promotes cell migration/invasion of CRC cells in vitro and formation of lung metastases in nude mice, which is attributed to the induction of epithelial-mesenchymal transition (EMT) by PCID2, thus favoring a mesenchymal phenotype with increased migratory and invasive potential [24–27]. The selective amplification of PCID2 in CRC patients, in conjunction with the compelling oncogenic function of PCID2 in multiple CRC models, collectively indicates a critical role of PCID1 in CRC progression and metastasis.

To further elucidate the molecular mechanism of PCID2 in CRC, we performed unbiased screening using immunoprecipitation-mass spectrometry and identified PML as a binding partner of PCID2. Direct interaction between PCID2 and PML was validated by co-immunoprecipitation and confocal immunofluorescence microscopy. Notably, PML has been reported to function as a tumor suppressor in many cancer types [14]. We next sought to evaluate the impact of PCID2-PML interaction. First, we observed dramatic reduction in PML protein expression without alteration in its corresponding mRNA in PCID2-overexpressing cells, which indicate that PCID2 regulates PML protein in a post-transcriptional manner. Indeed, ectopic PCID2 expression in CRC cells triggered polyubiquitination of PML, thereby accelerating its degradation via ubiquitin proteasome system (UPS). In agreement with our findings, PML has been shown to undergo proteasomal degradation in different cancers [17, 18, 28]. Next, we showed that silencing of PML abolished the pro-proliferative effect of PCID2 in CRC cell lines, inferring that oncogenic effect of PCID2 is dependent on PML. All these indicate that PCID2 mediates its oncogenic effect by directly interacting with PML and promoting the UPS-dependent degradation of PML.

To identify signaling cascade downstream of PCID2-PML interaction, we measured the activity of eight cancer-related pathways by luciferase reporter assay, revealing that PCID2 dramatically activated Top-flash (Wnt/β-catenin) but suppressed p53 signaling, which was also evidenced by enhanced c-Myc and decreased p53 protein, respectively. PML is known to form a complex with β-catenin and p300 to induce non-canonical β-catenin activation, whereby expression of tumor suppressive genes, such as ARF, are induced [16]. Consistently, we found that PCID2-PML axis modulates the balance between canonical and noncanonical β-catenin signaling in CRC cells. PCID2-induced degradation of PML repressed its interaction with β-catenin and non-canonical β-catenin signaling (e.g., ARF-p53). β-catenin, in turn, is released to engage canonical β-catenin signaling and drive expression of oncogenic factors c-Myc and cyclin D1. As a consequence, ARF and p53, downstream targets of noncanonical β-catenin signaling, were diminished in PCID2-expressing DLD1 and HCT116 cells, while PCID2 knockdown promoted their expression. Meanwhile, downstream genes of canonical Wnt/β-catenin signaling (Cyclin D1 and c-Myc) followed the opposite trend. Taken together, our results show that PCID2 promotes canonical Wnt/β-catenin signaling-driven tumorigenesis via the suppression of PML-mediated non-canonical β-catenin-ARF-p53 signaling.

**Fig. 7:**
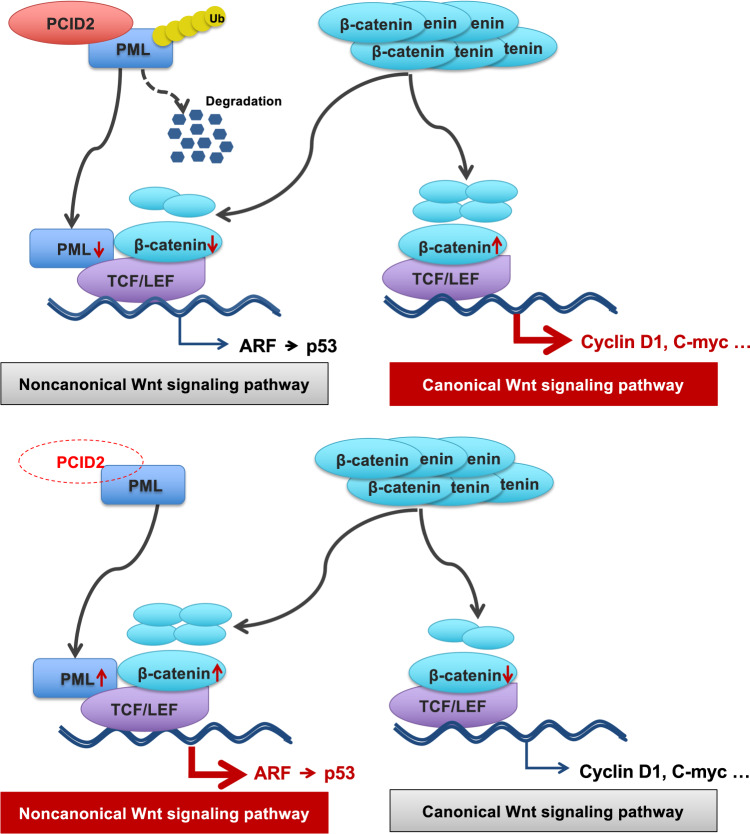
Proposed mechanistic scheme of PCID2 in promoting the canonical Wnt signaling pathway in colorectal cancer. PCID2 copy number amplification induces high expression of PCID2, and its amplification increased with colorectal cancer progression. Up-regulation of PCID2 suppressed PML by promoting its UPS-mediated degradation, which in turn, releases β-catenin to engage canonical Wnt/β-catenin signaling.

Our findings defined the molecular mechanism of PCID2 in promoting CRC and is summarized in Fig. 7. Under normal circumstances, PML competitively binds with β-catenin to promote non- canonical, tumor suppressive β-catenin signaling, while reducing with its involvement in canonical, oncogenic Wnt/β-catenin signaling. The up-regulation of PCID2 suppressed PML by promoting its UPS-mediated degradation, which in turn, releases β-catenin to engage canonical Wnt/β-catenin signaling, thus contributing to colorectal tumorigenesis.

In conclusion, our study first uncovered a novel amplification gene PCID2 in CRC. PCID2 promotes CRC tumorigenesis and metastasis in cell line and animal models, and it involved a PCID2-PML axis which promotes the participation of β-catenin in canonical, oncogenic Wnt/β-catenin pathway. Finally, PCID2 could serve as an independent predictive biomarker for recurrence in CRC patients.

## Materials and methods

### Clinical samples

In cohort I, surgically excised CRC tissues were collected form 114 CRC patients at Peking University Cancer Hospital. In cohort II, CRC and non-tumor colon tissues were obtained from 49 patients from Prince of Wales Hospital, the Chinese University of Hong Kong. In addition, TCGA database is containing 458 patients with colorectal cancer [29]. The patient demographics and clinicopathological features were shown in Supplementary Table 1. Besides, other two TCGA cohorts which include 376 and 543 CRC patents separately were enrolled in the study to evaluate the correlation of PCID2 amplification and clinical outcome. Written consent forms were obtained prior to tissue collection. The studies were approved by the Human Research Ethics Committee of The Chinese University of Hong Kong and Peking University Cancer Hospital.

### RNA extraction, semi-quantitative RT-PCR and real-time PCR analyses

Total RNA was extracted from cell pellets or tissues using QIAzol reagent (Qiagen, Valencia, CA) and cDNA was synthesized (Roche, Indianapolis, IN). Semi-quantitative RT-PCR was performed using Hot-star DNA polymerase (Invitrogen). Real-time PCR was performed using SYBR Green master mixture on HT7900 system (Applied Biosystems, Foster City, CA). Primer sequences are listed in Supplementary Table 2. Each sample was tested in triplicate. The fold change of gene expression level was determined by ΔΔCT method.

### Western blot analysis

Total protein was separated by sodium dodecyl sulfate-polyacrylamide gel electrophoresis (SDS-PAGE), and were transferred onto nitrocellulose membranes (GE Healthcare, Piscataway, NJ). The membrane was incubated with primary antibodies overnight, and then with secondary antibody at room temperature for 1 h. Proteins of interest were visualized using ECL Plus Western blotting Detection Reagents (GE Healthcare). The antibodies used are listed in Supplementary Table 3.

### In vivo tumorigenicity assay

For in vivo tumorigenicity assay, 1 × 10^6^ empty vector or PCID2-overexpressing cells were injected subcutaneously into dorsal right flank of 4-week-old male Balb/c nude mice. Tumor volume was measured every three days. Tumor volume (mm^3^) was estimated by measuring the longest and shortest diameter of the tumors (Formula: Volume = 0.5 × Length × 2 × Width). Mice were sacrificed at three weeks after injection. Tumors were excised and weighed. The excised tissues were either fixed in 10% neutral-buffered formalin or snap frozen in liquid nitrogen.

For in vivo metastasis assay, 1 × 10^6^ empty vector or shPCID2-transfected cells were injected into the lateral tail vein of 4-week-old male Balb/c nude mice. The nude mice were sacrificed after five weeks of injection. Tumors in lung were excised and weighed. The excised tissues were either fixed in 10% neutral-buffered formalin or snap frozen. All animal studies were approved by the Animal Experimentation Ethics Committee of The Chinese University of Hong Kong.

### Dual-luciferase reporter activity assay

Cells were transiently transfected with pcDNA3.1 empty vector, pcDNA3.1-PCID2, pCS2 + /Wnt-1 + pcDNA3.1 empty vector, pCS2 + /Wnt-1 + pcDNA3.1-PCID2 and co-transfected with TOPflash (0.2 µg/well) or FOPflash (0.2 µg/well) using lipofectamine 2000. Cells were harvested at 24 h post-transfection. Luciferase activity was measured by the Dual Luciferase Assay System (Promega, Madison, WI). The experiments were conducted three times in triplicates.

### Co-immunoprecipitation and mass spectrometry

HCT116 and DLD1 cells were transfected with pcDNA3.1 or pcDNA3.1-PCID2. Total proteins were extracted by CytoBuster Protein Extraction Reagent (Novagen, Darmstadt, Germany) with Protease inhibitor. For immunoprecipitation, 300 ug of precleared cell lysate was incubated with 30 μl of protein G PLUS-Agarose (Santa Cruz Biotechnology, Santa Cruz, CA) for overnight at 4 °C. Immunoprecipitated complexes were eluted with 2 × SDS-PAGE loading buffer. Candidate proteins were separated by SDS-PAGE and identified by mass spectrometry.

### Ubiquitination assay

DLD1 and HCT116 cells were transfected with pcDNA3.1-PCID2 or empty vector and incubated in the presence or absence of 30 μM MG132 (Cell Signaling Technology, Danvers, Massachusetts, USA) for 24 h. Total proteins were extracted by using CytoBuster Reagent, followed by immuno-precipitation with anti-PML or anti-IgG. Immunoprecipitated complexes were detected by western blot using anti-ubiquitin. The inputs were quantified by western blot analysis with antibodies against PML and GAPDH, respectively.

### CTNNB1 protein pull down assay

The total proteins were extracted from DLD1-vecter, DLD1-PCID2, HCT116-shNC and HCT116-shPCID2 cells, incubated with CTNNB1 antibody and immunoprecipitated with protein G PLUS-Agarose beads overnight at 4 °C. The beads with protein complexes were washed, separated by SDS-PAGE and visualized by western blot with antibodies against PML and LEF1.

### Statistical analysis

All results were expressed as mean ± SD. The Mann–Whitney *U* test was performed to compare the difference in PCID2 protein expression between tumor and adjacent nontumor tissues. Differences in tumor growth rate between the two groups of nude mice was determined by repeated-measures analysis of variance. The χ2 test was used for comparison of patient characteristics and distributions of PCID2 expression and covariates by vital status. Crude risk ratios (RRs) of recurrence associated with PCID2 expression and other predictor variables were estimated by using a univariate Cox proportional hazards regression model. A multivariate Cox model was constructed to estimate the adjusted RR for PCID2 expression. The overall recurrence rate in relation to methylation status was evaluated by the recurrence curve and the log-rank test. Analysis of overall recurrence was limited to a 5-year period to avoid the probability of the recurrence not being related to CRC. A *p* < 0.05 was regarded as statistically significant.

## Supplementary information

Supplementary materials

Supplementary Fig. 1

Supplementary Fig. 2

Supplementary Fig. 3

Supplementary Fig. 4

Supplementary Fig. 5

Supplementary Fig. 6

Supplementary Fig. 7

Supplementary Fig. 8

Supplementary Table 1

Supplementary Table 2

Supplementary Table 3

Supplementary Table 4
