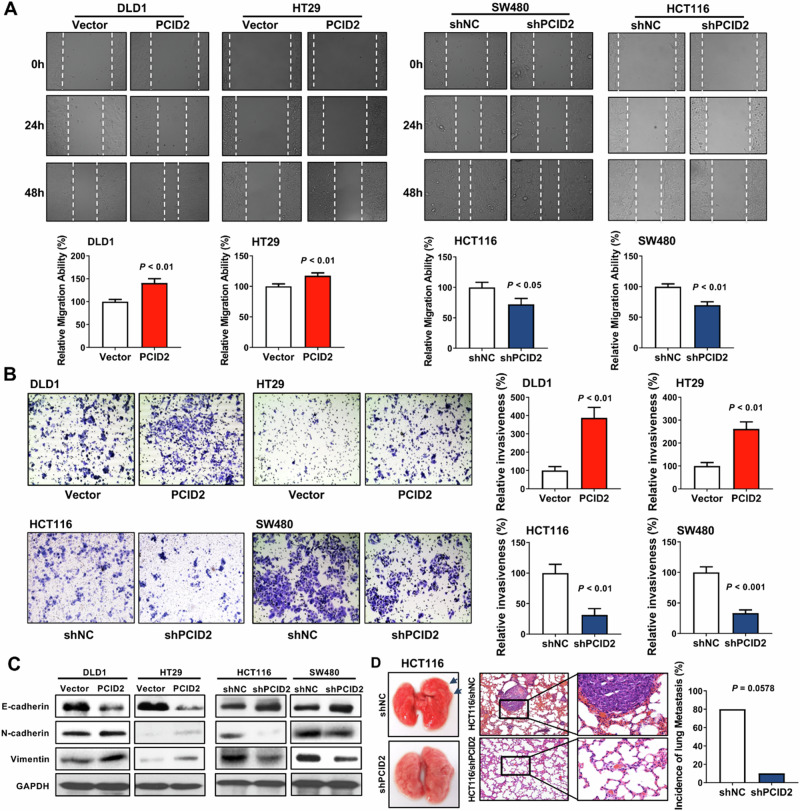# Correction: A novel amplification gene PCI domain containing 2 (PCID2) promotes colorectal cancer through directly degrading a tumor suppressor promyelocytic leukemia (PML)

**DOI:** 10.1038/s41388-025-03304-4

**Published:** 2025-02-12

**Authors:** Jingwan Zhang, Jianning Zhai, Chi Chun Wong, Huarong Chen, Xiaohong Wang, Jiafu Ji, Jun Yu

**Affiliations:** 1 Institute of Digestive Disease and Department of Medicine and Therapeutics, State Key Laboratory of Digestive Disease, Li Ka Shing Institute of Health Sciences, Shenzhen Research Institute, The Chinese University of Hong Kong, Hong Kong, China; 2 Department of Gastrointestinal Oncology, Peking University Cancer Hospital & Institute, Beijing, China

Correction to: *Oncogene* 10.1038/s41388-021-01941-z, published online 8 October 2021

Following the publication of this article an error was noted in Figure 4B. The authors inadvertently used incorrect images for the HCT116 cells. The updated version of Figure 4B (HCT116) is shown below. The authors confirm that the conclusions of the article are not affected by this correction and apologize for any inconvenience this may have caused. The original article has been corrected.

Former version:
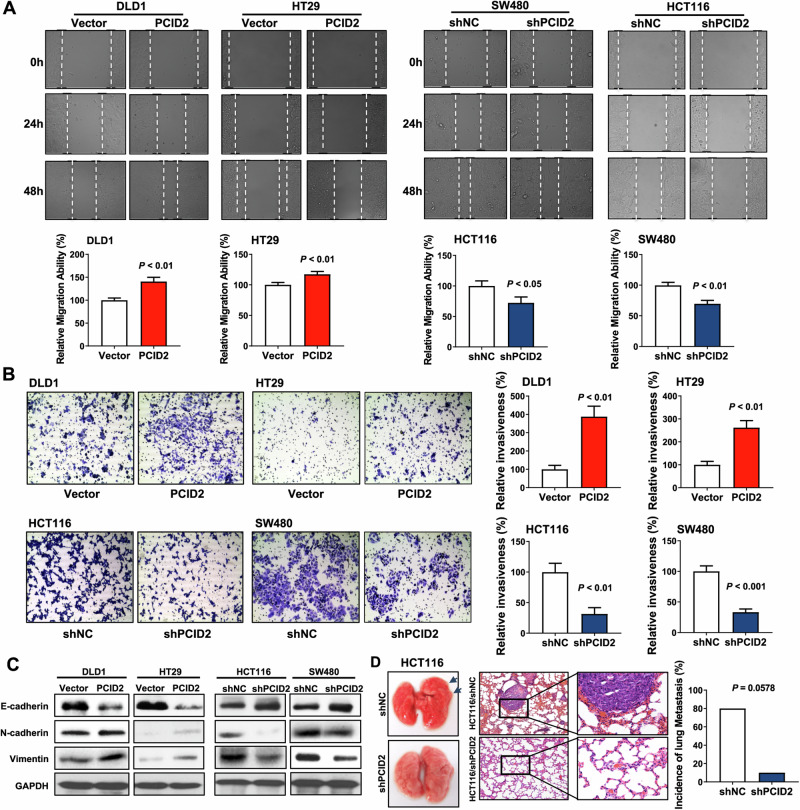


Correct version: