# Supplementary material for: A novel amplification gene PCI domain containing 2 (PCID2) promotes colorectal cancer through directly degrading a tumor suppressor promyelocytic leukemia (PML)
Source: Oncogene. 2021 Oct 8;40(49):6641–52. doi: 10.1038/s41388-021-01941-z (PMC8660639; doi:10.1038/s41388-021-01941-z)
Supplement: Supplementary file 1 — Supplementary materials [file 41388_2021_1941_MOESM1_ESM.docx]

**SUPPLEMENTARY MATERIAL**

**CRC cell lines**

CRC cell lines (DLD1, HCT116, HT-29, LOVO, SW480, SW620, and SW1116) were obtained from the American Type Culture Collection (ATCC, Manassas, VA). RPMI 1640 medium (Gibco BRL, Rockville, MD) was used except for HCT116 that used McCoy’s 5A medium (Gibco BRL). All the cell lines were cultured at 37°C in a humidified atmosphere of 10% CO_2_.

**Construction of PCID2 expression plasmid and establishment of stable cells**

The full-length open reading frame of PCID2 was amplified by normal human colon cDNA and subcloned into the mammalian expression vector pcDNA3.1 (Thermo Fisher Scientific, Waltham, MA). HT29 or DLD1 cells were transfected with pcDNA3.1 or pcDNA3.1-PCID2 plasmid using lipofectamine 2000 (Life Technologies) and selected for 2 weeks with G418 to obtain stable cell lines with PCID2 overexpression.

**RNA interference and transfection**

PCID2 knockdown in HCT116 and SW480 cell lines was performed by a shRNA targeting PCID2. Stable PCID2 knockdown cells were obtained after puromycin selection for 2 weeks. siRNA against PML (F: 5’-CCCGCAAGACCAACAACAUTT-3’; R: 5’-AUGUUGUUGGUCUUGCGGGTT-3’) was purchased (Life Technologies). 10 nM PML siRNA was used to transfect HCT116 and DLD1 cells using lipofectamine 2000.

**Immunohistochemistry**

Immunohistochemistry was performed on 5 um paraffin sections. Following deparaffinization and rehydration, antigen retrieval was performed using citrate buffer (pH 6.0) for 20 min, followed by incubation with anti-PCID2 antibody (Novus Biologicals, Littleton, CO) with dilution of 1:200 at room temperature. PCID2 staining was scored according to percentage of positive tumor cells (0, none; 1, < 20% of positive staining cells; 2, 20-50% of positive staining cells; 3, > 50% of positive staining cells).

**Cell viability and colony formation assay**

Cell viability of stably transfected cells was examined using the Vybrant MTT Cell Proliferation Assay Kit (Life Technologies). For colony formation, colonies were fixed with methanol and stained with 0.5% crystal violet solution. Colonies with more than 50 cells were counted. All experiments were conducted three times in triplicates.

**Cell cycle analysis**

CRC cells were collected by trypsinization and fixed in 70% ethanol-PBS overnight. The cells were then stained in propidium iodide (50 μg/ml) (BD Pharmingen, Franklin Lakes, NJ) and analyzed by FACSCalibur (BD Biosciences, San Diego, CA). For each experiment, 10,000 events were counted. Cell-cycle profiles were then analyzed by ModFit 3.0 software (BD Biosciences).

**Apoptosis assay**

Cell apoptosis was determined by staining cells with Annexin V and 7-amino-actinomycin (7- AAD) (BD Biosciences) with subsequent flow cytometry analysis. Apoptosis profiles were determined by ModFit 3.0 software (BD Biosciences).

**Wound-healing assay**

CRC cell lines were cultured in six-well plates. When cells reached 80% confluence, three wounds in each well were created by a sterile plastic scratcher. Fresh medium with 5% fetal bovine serum was then replaced, and wound healing was observed for 48h.

**Invasion assay**

CRC cell lines in serum free medium were placed in the upper chamber of BD BioCoat Matrigel Invasion Chamber (BD Biosciences)**.** Complete culture medium was used as the chemoattractant up the lower chamber. After 48 hours, cells that passed through the insert membrane were stained with 0.5% crystal violet. Invaded cells were counted under an inverted microscope and photographed.

**Ki-67 and TUNEL staining**

Ki-67 was detected in paraffin-embedded subcutaneous tumor tissue using an avidin-biotin complex immunoperoxidase method (Abcam, Chambridge, MA). Proliferation index was evaluated by the percentage of Ki-67 positive staining cells. Terminal deoxynucleotidyl transferase-mediated nick-end labeling (TUNEL) assay was performed to determine apoptosis. Apoptosis index was calculated as the percentage of TUNEL-positive nuclei after counting at least 1000 cells.

**Polymerase Chain Reaction Array**

Cancer Pathway Finder PCR Array (PAHS-033Z) was used.
