# Supplementary material for: A novel amplification gene PCI domain containing 2 (PCID2) promotes colorectal cancer through directly degrading a tumor suppressor promyelocytic leukemia (PML)
Source: Oncogene. 2021 Oct 8;40(49):6641–52. doi: 10.1038/s41388-021-01941-z (PMC8660639; doi:10.1038/s41388-021-01941-z)

Supplementary Fig.1: mRNA expression of PCID2 was not related to CRC stages in Cohort I and TCGA Cohort. Two-tailed unpaired t test.

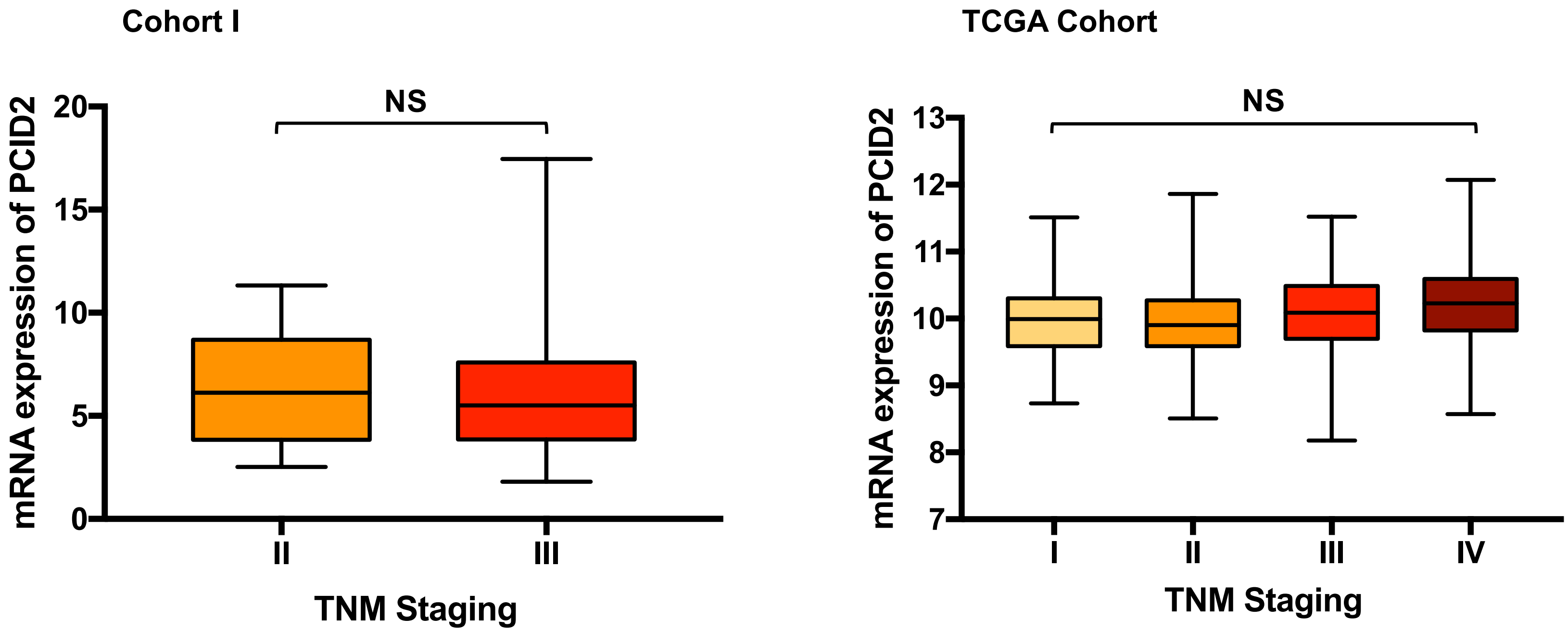

Supplement: Supplementary file 2 — Supplementary Fig. 1 [file 41388_2021_1941_MOESM2_ESM.pdf]
