# Supplementary figures and images for: A novel amplification gene PCI domain containing 2 (PCID2) promotes colorectal cancer through directly degrading a tumor suppressor promyelocytic leukemia (PML)
Source: Oncogene. 2021 Oct 8;40(49):6641–52. doi: 10.1038/s41388-021-01941-z (PMC8660639; doi:10.1038/s41388-021-01941-z)

**Supplementary Fig. 2:** The proportion of PCID2 copy number variation in different types of cancer.

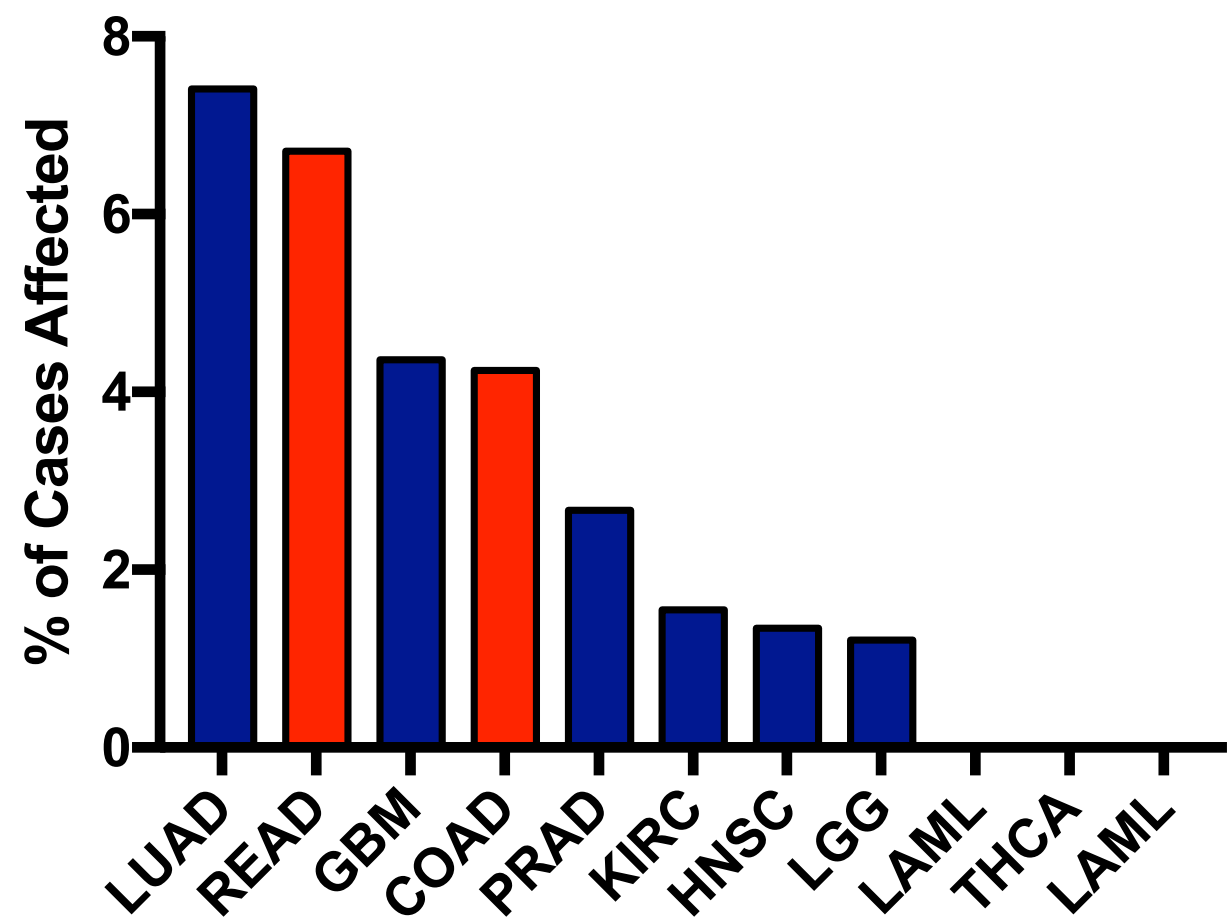

Supplement: Supplementary file 3 — Supplementary Fig. 2 [file 41388_2021_1941_MOESM3_ESM.pdf]
