# Supplementary material for: A novel amplification gene PCI domain containing 2 (PCID2) promotes colorectal cancer through directly degrading a tumor suppressor promyelocytic leukemia (PML)
Source: Oncogene. 2021 Oct 8;40(49):6641–52. doi: 10.1038/s41388-021-01941-z (PMC8660639; doi:10.1038/s41388-021-01941-z)

Supplementary Fig. 3: Overexpression and knockdown efficiency of PCID2 in CRC cells were confirmed by RT-PCR and Western blot.

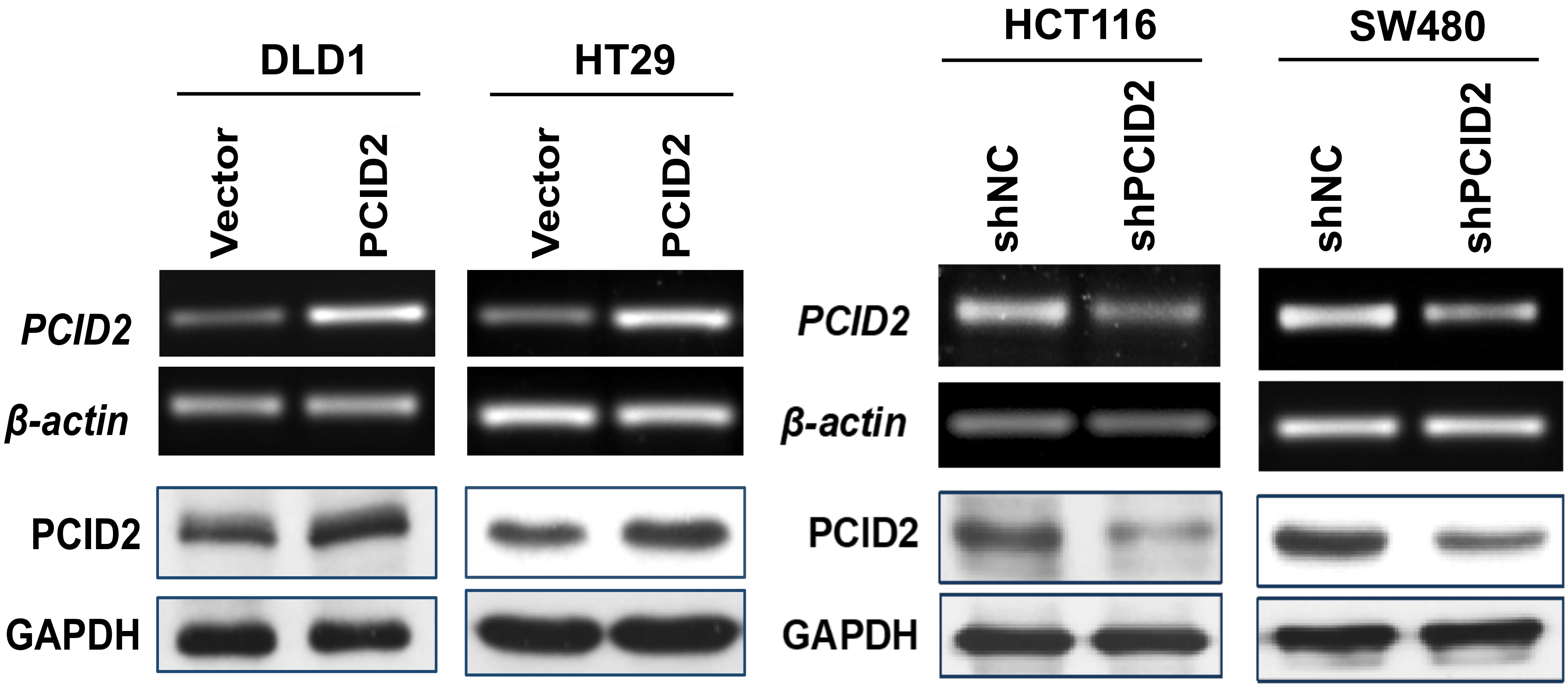

Supplement: Supplementary file 4 — Supplementary Fig. 3 [file 41388_2021_1941_MOESM4_ESM.pdf]
