# Supplementary material for: A novel amplification gene PCI domain containing 2 (PCID2) promotes colorectal cancer through directly degrading a tumor suppressor promyelocytic leukemia (PML)
Source: Oncogene. 2021 Oct 8;40(49):6641–52. doi: 10.1038/s41388-021-01941-z (PMC8660639; doi:10.1038/s41388-021-01941-z)

Supplementary Fig. 4: Regulated downstream targets of PCID2 identified by Cancer Pathway PCR Array.

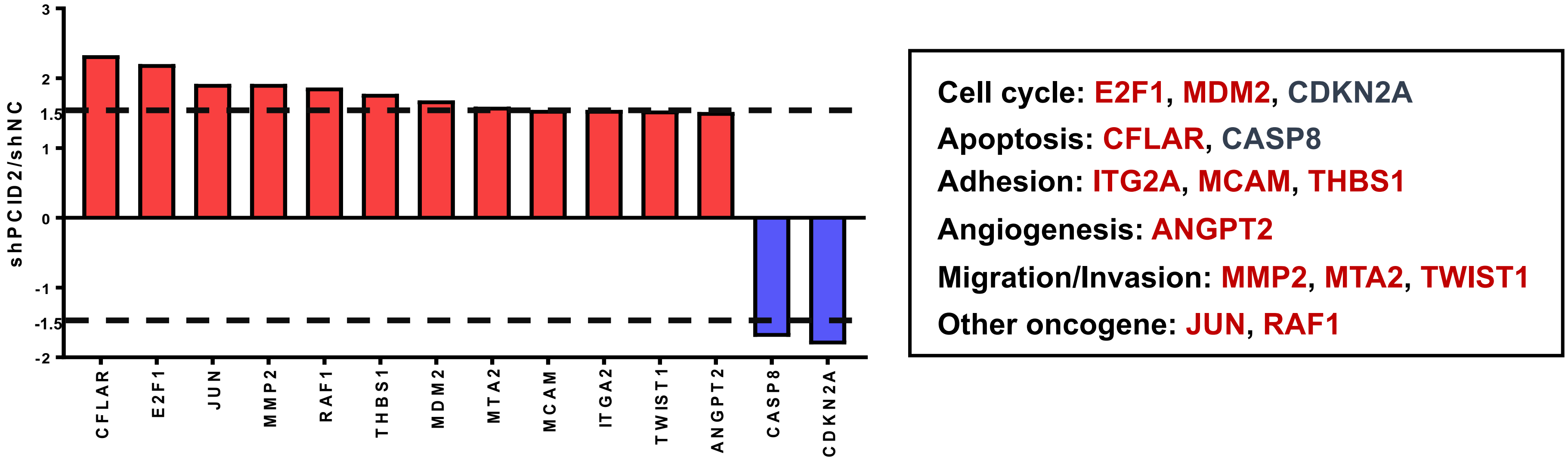

Supplement: Supplementary file 5 — Supplementary Fig. 4 [file 41388_2021_1941_MOESM5_ESM.pdf]
