# Supplementary material for: A novel amplification gene PCI domain containing 2 (PCID2) promotes colorectal cancer through directly degrading a tumor suppressor promyelocytic leukemia (PML)
Source: Oncogene. 2021 Oct 8;40(49):6641–52. doi: 10.1038/s41388-021-01941-z (PMC8660639; doi:10.1038/s41388-021-01941-z)

Supplementary Fig. 5: A PCID2-overexpressed DLD1 cells and PCID2-depleted HCT116 cells promoted and inhibited tumor volume in nude mice respectively. **B** PCID2 expression in mice tumors were confirmed at mRNA and protein levels.

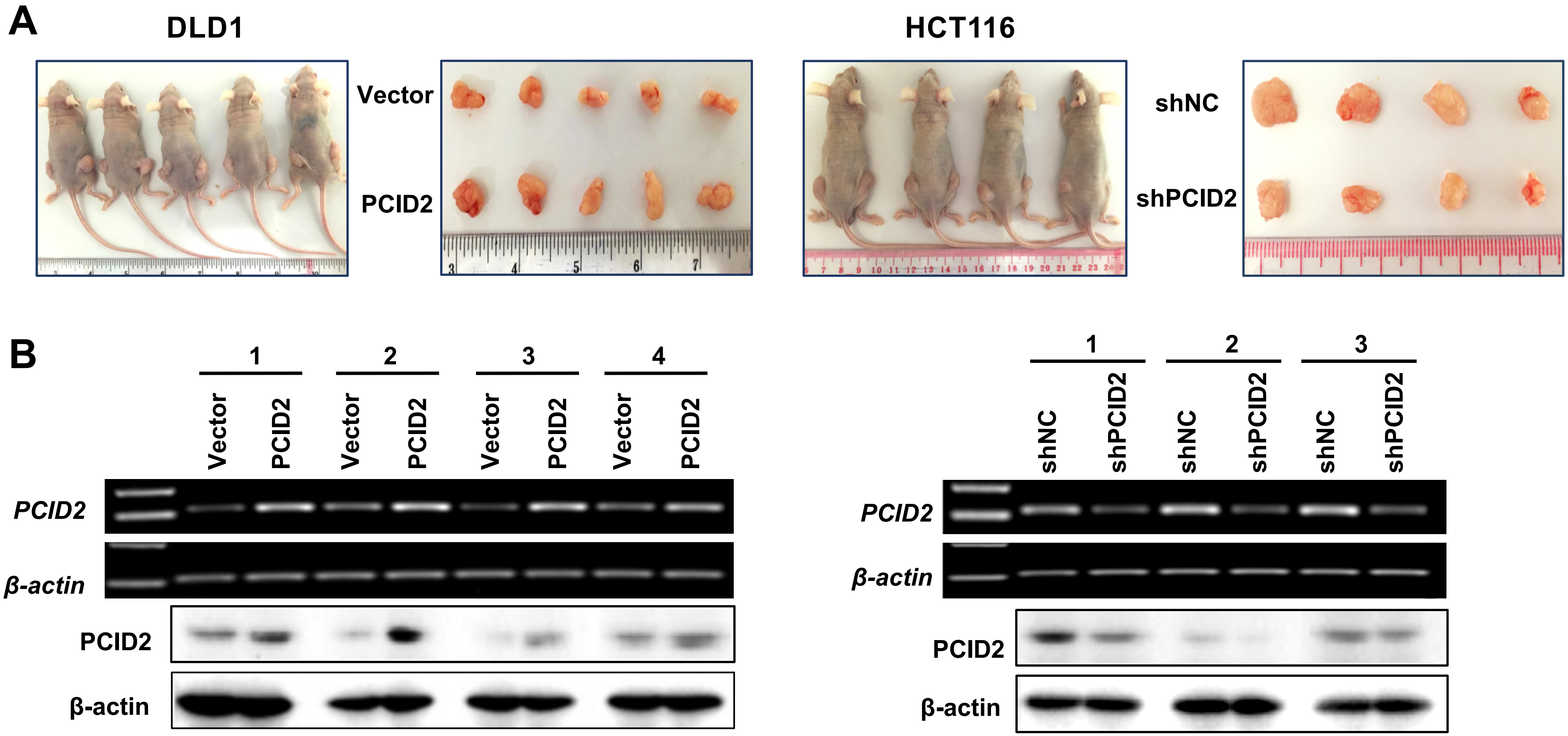

Supplement: Supplementary file 6 — Supplementary Fig. 5 [file 41388_2021_1941_MOESM6_ESM.pdf]
