# Supplementary material for: A novel amplification gene PCI domain containing 2 (PCID2) promotes colorectal cancer through directly degrading a tumor suppressor promyelocytic leukemia (PML)
Source: Oncogene. 2021 Oct 8;40(49):6641–52. doi: 10.1038/s41388-021-01941-z (PMC8660639; doi:10.1038/s41388-021-01941-z)

Supplementary Fig. 7: Recurrence curves showed that the patients with CRC with low PML expression had increased risk of recurrence compared with those with high PCID2 (*P* < 0.05 for TCGA cohort, log-rank test)**.** log-rank test.

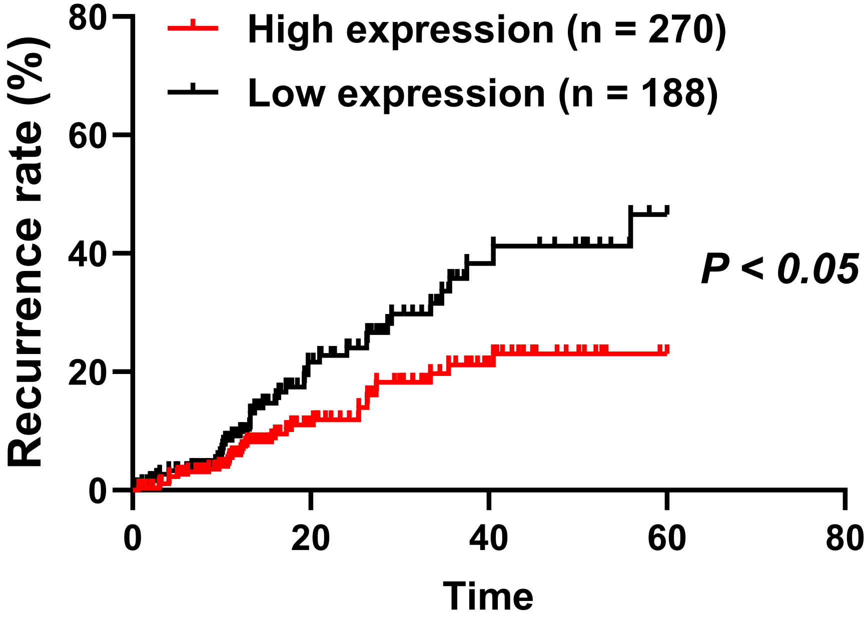

Supplement: Supplementary file 8 — Supplementary Fig. 7 [file 41388_2021_1941_MOESM8_ESM.pdf]
