# Supplementary material for: A novel amplification gene PCI domain containing 2 (PCID2) promotes colorectal cancer through directly degrading a tumor suppressor promyelocytic leukemia (PML)
Source: Oncogene. 2021 Oct 8;40(49):6641–52. doi: 10.1038/s41388-021-01941-z (PMC8660639; doi:10.1038/s41388-021-01941-z)
Supplement: Supplementary file 10 — Supplementary Table 1 [file 41388_2021_1941_MOESM10_ESM.docx]

| **Cohort I** |  |  |  |  |
| --- | --- | --- | --- | --- |
|  | **High expression (n=38)** | **%** | **Low expression (n=73)** | **%** |
| **Age** | 60.9±14.5 |  | 57.7±13.4 |  |
| **Gender** |  |  |  |  |
| M | 22 | 31.9 | 47 | 68.1 |
| F | 16 | 38.1 | 26 | 61.9 |
| **T stage** |  |  |  |  |
| II | 3 | 7.9 | 10 | 13.7 |
| III | 35 | 92.1 | 63 | 86.3 |
| **Localization** |  |  |  |  |
| Colon | 14 | 28 | 36 | 72 |
| Rectum | 24 | 39.3 | 37 | 60.7 |
| **Micorsatellite status** |  |  |  |  |
| Moderate/High | 35 | 46.1 | 41 | 53.9 |
| Low | 3 | 12 | 22 | 88 |
| **Cohort II** |  |  |  |  |
|  | **High expression (n=13)** | **%** | **Low expression (n=36)** | **%** |
| **Age** | 63.9±8.5 |  | 66.8±12.7 |  |
| **Gender** |  |  |  |  |
| M | 4 | 14.3 | 24 | 85.7 |
| F | 9 | 42.9 | 12 | 57.1 |
| **TNM** |  |  |  |  |
| I | 1 | 14.3 | 6 | 85.7 |
| II | 4 | 21.1 | 15 | 78.9 |
| III | 8 | 34.8 | 15 | 65.2 |
| **Localization** |  |  |  |  |
| Colon | 1 | 25 | 3 | 75 |
| Rectum | 12 | 26.7 | 33 | 73.3 |
| **Cohort III (TCGA)** |  |  |  |  |
|  | **High expression (n=202)** | **%** | **Low expression (n=256)** | **%** |
| **Age** | 65.2±11.5 |  | 64.9±13.4 |  |
| **Gender** |  |  |  |  |
| M | 117 | 45 | 143 | 55 |
| F | 85 | 42.9 | 113 | 57.1 |
| **TNM** |  |  |  |  |
| I | 36 | 43.4 | 47 | 56.6 |
| II | 58 | 34.1 | 112 | 65.9 |
| III | 63 | 45.7 | 75 | 54.3 |
| IV | 45 | 68.2 | 21 | 31.8 |
| **Localization** |  |  |  |  |
| Colon | 144 | 41.1 | 206 | 58.9 |
| Rectum | 58 | 53.7 | 50 | 46.3 |
| **Micorsatellite status** |  |  |  |  |
| MSI-L/MSS | 194 | 49.1 | 201 | 50.9 |
| MSI-H | 8 | 12.7 | 55 | 87.3 |

**Supplementary Table 1.** Clinicopathological characteristics and PCID2 expression in tumor tissues of colon cancer patients.
