# Supplementary material for: A novel amplification gene PCI domain containing 2 (PCID2) promotes colorectal cancer through directly degrading a tumor suppressor promyelocytic leukemia (PML)
Source: Oncogene. 2021 Oct 8;40(49):6641–52. doi: 10.1038/s41388-021-01941-z (PMC8660639; doi:10.1038/s41388-021-01941-z)
Supplement: Supplementary file 11 — Supplementary Table 2 [file 41388_2021_1941_MOESM11_ESM.docx]

| p53 | F | GCCCAACAACACCAGCTCCT |
| --- | --- | --- |
|  | R | CCTGGGCATCCTTGAGTTCC |
| ARF(p14) | F | GCCAGTGTCCTTCCACCTGTC |
|  | R | GCCTCGTTCACACGCTCTCTG |
| CCND1(CyclinD1) | F | CCCTCGGTGTCCTACTTCAA |
|  | R | AGGAAGCGGTCCAGGTAGTT |
| PML | F | GTCTCCAAGTGCCTCACTC |
|  | R | GGCTTCACCGTCAGCGTA |
| β−actin | F | GCATGGGTCAGAAGGATTCCT |
|  | R | TCGTCCCAGTTGGTGACGAT |
| c-Myc | F | CAAGAGGCGAACACACAACG |
|  | R | GTCGTTTCCGCAACAAGTCC |
| PCID2 | F | CAGAAGCTGGTGGTCAGCAA |
|  | R | GGCTCCGTGTACTTTCAACACA |

**Supplementary Table 2.** Primer list
