# Supplementary material for: A novel amplification gene PCI domain containing 2 (PCID2) promotes colorectal cancer through directly degrading a tumor suppressor promyelocytic leukemia (PML)
Source: Oncogene. 2021 Oct 8;40(49):6641–52. doi: 10.1038/s41388-021-01941-z (PMC8660639; doi:10.1038/s41388-021-01941-z)
Supplement: Supplementary file 12 — Supplementary Table 3 [file 41388_2021_1941_MOESM12_ESM.docx]

|  |  |  |
| --- | --- | --- |
| Caspase-3 | Cell signaling | 9665s |
| Cleaved Caspase-3 | Cell signaling | 9661s |
| Caspase-7 | Cell signaling | 9492s |
| Cleaved Caspase-7 | Cell signaling | 9491s |
| Caspase-8 | Cell signaling | 9746s |
| cleaved caspase 8 | Cell signaling | 9496s |
| Cleaved PARP | Cell signaling | 5625 |
| PARP | Cell signaling | 9532 |
| Cyclin D1 | Santa Cruz | sc-246 |
| p21 (F-5) | Santa Cruz | sc-6246 |
| E-Cadherin (4A2) | Cell signaling | 14472 |
| N-Cadherin | cell signalling | 4061s |
| Vimentin (D21H3) | cell signalling | #5741 |
| p53 | Santa Cruz | sc-126x |
| c-Myc | Cell signaling | 9402 |
| PCID2 antibody | GeneTex | GTX52023 |
| PML (PG-M3) | Santa Cruz | sc-966 |
| LEF1 | cell signalling | 2230 |
| beta-catenin (H-102) | Santa Cruz | sc-7199 |
| active beta-catenin | Millipore | 05-665 |
| GAPDH | Santa Cruz | sc-47724 |

**Supplementary Table 3.** Antibody list
