# Supplementary material for: A novel amplification gene PCI domain containing 2 (PCID2) promotes colorectal cancer through directly degrading a tumor suppressor promyelocytic leukemia (PML)
Source: Oncogene. 2021 Oct 8;40(49):6641–52. doi: 10.1038/s41388-021-01941-z (PMC8660639; doi:10.1038/s41388-021-01941-z)
Supplement: Supplementary file 13 — Supplementary Table 4 [file 41388_2021_1941_MOESM13_ESM.docx]

| **Cox regression analysis of potential recurrence predictor for patients with colon cancer (cohort III)** | | | | | |
| --- | --- | --- | --- | --- | --- |
|  | **Univariate Cox regression analysis** | |  | **Multivariate Cox regression analysis** | |
| **Variables** | **RR (95% CI)** | ***P* value** |  | **RR (95% CI)** | ***P* value** |
| Age | **0**.995 (0.976 to 1.014) | 0.582 |  | 0.995 (0.975 to 1.015) | 0.619 |
| Gender |  |  |  |  |  |
| M | 0.684 (0.431 to 1.085) | 0.107 |  | 0.736 (0.457 to 1.187) | 0.208 |
| F | 1 |  |  | 1 |  |
| TNM |  |  |  |  |  |
| I | 0.136 (0.051 to 0.363) | <0.001 |  | 0.158 (0.059 to 0.427) | <0.001 |
| II | 0.258 (0.142 to 0.471) | <0.001 |  | 0.324 (0.173 to 0.606) | <0.001 |
| III | 0.370 (0.202 to 0.679) | <0.001 |  | 0.424 (0.229 to 0.786) | 0.006 |
| IV | 1 |  |  | 1 |  |
| Localisation |  |  |  |  |  |
| Colon | 1.204 (0.722 to 2.007) | 0.477 |  |  |  |
| Rectum | 1 |  |  |  |  |
| Microsatellite status |  |  |  |  |  |
| MSI-L/MSS | 0.608 (0.279 to 1.324) | 0.210 |  |  |  |
| MSI-H | 1 |  |  |  |  |
| PCID2 expression |  |  |  |  |  |
| High | 1.913 (1.280 to 2.860) | 0.002 |  | 1.569 (1.037 to 2.376) | 0.033 |
| Low | 1 |  |  | 1 |  |

**Supplementary Table 4.** Cox regression analysis (TCGA dataset)
